# Comparison of survival of patients with metastases from known versus unknown primaries: survival in metastatic cancer

**DOI:** 10.1186/1471-2407-13-36

**Published:** 2013-01-28

**Authors:** Matias Riihimäki, Hauke Thomsen, Akseli Hemminki, Kristina Sundquist, Kari Hemminki

**Affiliations:** 1 Division of Molecular Genetic Epidemiology, German Cancer Research Centre (DKFZ), 69120, Heidelberg, Germany; 2 Center for Primary Health Care Research, Lund University, Malmö, Sweden; 3 Cancer Gene Therapy Group, Molecular Cancer Biology Program & Transplantation Laboratory & Haartman Institute, University of Helsinki, 00290, Helsinki, Finland; 4 Stanford Prevention Research Center, Stanford University School of Medicine, California, CA, USA

**Keywords:** Metastasis, Cancer survival, Regression analysis, Cancer of unknown primary, CUP

## Abstract

**Background:**

Cancer of unknown primary site (CUP) is considered an aggressive metastatic disease but whether the prognosis differs from metastatic cancers of known primary site is not known. Such data may give insight into the biology of CUP and the metastatic process in general.

**Methods:**

6,745 cancer patients, with primary metastatic cancer at diagnosis, were identified from the Swedish Cancer Registry, and were compared with 2,881 patients with CUP. Patients were diagnosed and died between 2002 and 2008. The influence of the primary site, known or unknown, on survival in patients with metastases at specific locations was investigated. Hazard ratios (HRs) of death were estimated for several sites of metastasis, where patients with known primary sites were compared with CUP patients.

**Results:**

Overall, patients with metastatic cancers with known primary sites had decreased hazards of death compared to CUP patients (HR = 0.69 [95% CI = 0.66–0.72]). The exceptions were cancer of the pancreas (1.71 [1.54–1.90]), liver (1.58 [1.36–1.85]), and stomach (1.16 [1.02–1.31]). For individual metastatic sites, patients with liver or bone metastases of known origin had better survival than those with CUP of the liver and bone. Patients with liver metastases of pancreatic origin had an increased risk of death compared with patients with CUP of the liver (1.25 [1.06–1.46]). The median survival time of CUP patients was three months.

**Conclusions:**

Patients with CUP have poorer survival than patients with known primaries, except those with brain and respiratory system metastases. Of CUP sites, liver metastases had the worst prognosis. Survival in CUP was comparable to that in metastatic lung cancer. The aggressive behavior of CUP may be due to initial immunosuppression and immunoediting which may allow accumulation of mutations. Upon escape from the suppressed state an unstoppable tumor spread ensues. These novel data on the epidemiology of the metastatic process at the population level demonstrated large survival differences in organ defined metastases depending on the original cancer.

## Background

Cancer possesses, by definition, the potential to metastasize and most cancer patients die of metastasis, against which even modern therapies often feature limited utility [1,2]. A large proportion of fatal cancers, such as pancreatic, liver, lung and stomach cancers present with distant metastasis at diagnosis. For these and other cancers the size of the primary tumor shows some correlation with the likelihood of detecting distant metastasis [3,4]. The TNM classification, considering the size of the primary tumor and metastasis to lymph nodes or distant sites, provides a prognostic prediction of the outcome even though the classification does not consider the organs of metastatic growth. The lack of therapeutic success against cancer metastasis is partially due to the limited understanding of the metastatic process [4-6]. Epidemiological approaches to metastatic cancer are hampered by the fact that cancer registries almost invariably only consider the primary tumor and the presence or absence of metastasis can only be concluded from the TNM status, if recorded by the registry. Consequently, population-based survival studies are usually not able to consider the site of metastasis and even death certificates give the primary cancer as an underlying cause of death. Hospital discharge data have been used to obtain information on metastasis in the treated patients but there may be concerns about coverage and accuracy even if nation-wide data were available, as through the Medicare system in the USA and the national Hospital Discharge Register in Sweden [7,8]. Data on metastatic sites may be recorded in hospital-based registers or in clinical trials but these may not be generalizable to the population at large [9-11].

We have found two possibilities to access metastasis data at a nation-wide level. International Classification of Diseases (ICD) version 9 and subsequent versions provide information on metastatic sites as multiple causes of death, available at the Swedish Causes of Death Register. These data are highly reliable [12], partially because of the high proportion of deaths take place at hospitals [13], but also due to the fact that almost 95% of death certificates in cancer patients are based on examination at hospital prior to death [14]. We use here this source in combination with the TNM data to define survival in common cancers based on the site of metastasis. The other source to metastatic cancer is through cancer of unknown primary site (CUP). For CUP, ICD-9 indicates the location of metastasis according to affected lymph nodes or organ sites, because the primary tumor is not known [7]. Furthermore, according to the Swedish Causes of Death Register, the underlying cause of death for CUP patients is usually the cancer of the organ system that has killed the patient, i.e., the fatal metastatic site, as judged by the death registrar. This practice is different from all other cancers but it will provide useful information for the study of the metastatic process.

In this study we compare survival times for organ-specific metastasis from known primary cancers and from CUP. We hypothesize, in view of the aggressive behavior of CUP, that CUP patients have a worse survival than those with metastatic cancers with known primaries. A counter-hypothesis posits that the survival may be better for CUP because the immunological or other mechanisms that forced the involution of the primary tumors in CUP might also control the spread of metastasis [15]. CUP patients with brain metastasis have indeed survived better than those with other brain metastases [8,16]. However, the data are lacking for extracranial sites and the present study provides novel population-based results for all common cancers and their metastases.

## Methods

Cancers were identified from the Swedish Cancer Registry, which is based on the compulsory notification of cancer cases. Cancer cases in the Database were coded according to ICD-7 since 1958, ICD-9 since 1987 and ICD-10 since 1993. ICD-9 and ICD-10 codes are translated back to ICD-7. However, only ICD-9 and ICD-10 coding allow identification of the site of CUP. Causes of death were obtained from the Swedish Cause of Death Registry, which used ICD-10 coding since 1997 [17]. National Census Data were included into the dataset to obtain information on socioeconomic status and geographical location of residence on each individual. Linking the databases was made possible by using the national 10-digit civic identification number, given to each person in Sweden for his or her lifetime. In order to provide anonymity, the identification number has been replaced by a serial number for each person.

We investigated survival in eight common metastatic cancers, which were identified by their ICD-7 codes: lung cancer (162,163), colorectal cancer (153,154.0), prostate cancer (177, only men), pancreatic cancer (157), liver cancer (155), stomach cancer (151), breast cancer (170, only women), kidney cancer (180), and bladder cancer (181.0). ICD-10 codes used in identifying metastases from death certificates and CUPs from the cancer registry are discussed below. When a CUP diagnosis is made, the metastases are often already present in multiple locations [15]. Inclusion of CUP patients with only one distant metastasis at diagnosis was desired. Therefore, CUP patients with or more than one primary cancer or one remark of metastasis in the death certificate were excluded from the reference (CUP) group. Furthermore, we also restricted all included cancer cases to those with 1) positive distant metastasis status upon diagnosis and 2) only one mentioned metastasis in their death certificate. Thus, we may assume that specific metastases mentioned in death certificate were present at diagnosis. ICD-10 codes used for identifying CUP locations were the following: Any location (C76, C78, C79), brain (C79.3), bone (C79.5), gastro-intestinal (C78.4-8, except .7), liver (C78.7), and respiratory system (C78.0-3).

Follow-up of cancer patients started at diagnosis, and terminated at death. Because the TNM classification has been available since 2002, analyses were restricted to patients receiving a cancer diagnosis during 2002 or later. All deaths occurred until the end of 2008. Only patients that survived at least one month were included. A Cox regression hazard model was used to calculate HRs for death, where cancer patients with metastasis upon diagnosis (M1) with known primary sites were compared with a reference group, encompassing patients with CUP with defined organ metastasis. Age (months) was the underlying time scale. The statistical model included socio-economic index, gender and geographical location of residence as covariates. All calculations were performed with SAS-software (PROC PHREG; SAS Version 9.2; SAS Institute, Cary, NC).

The study was approved by the ethical committee of Lund University.

## Results

**Figure 1:**
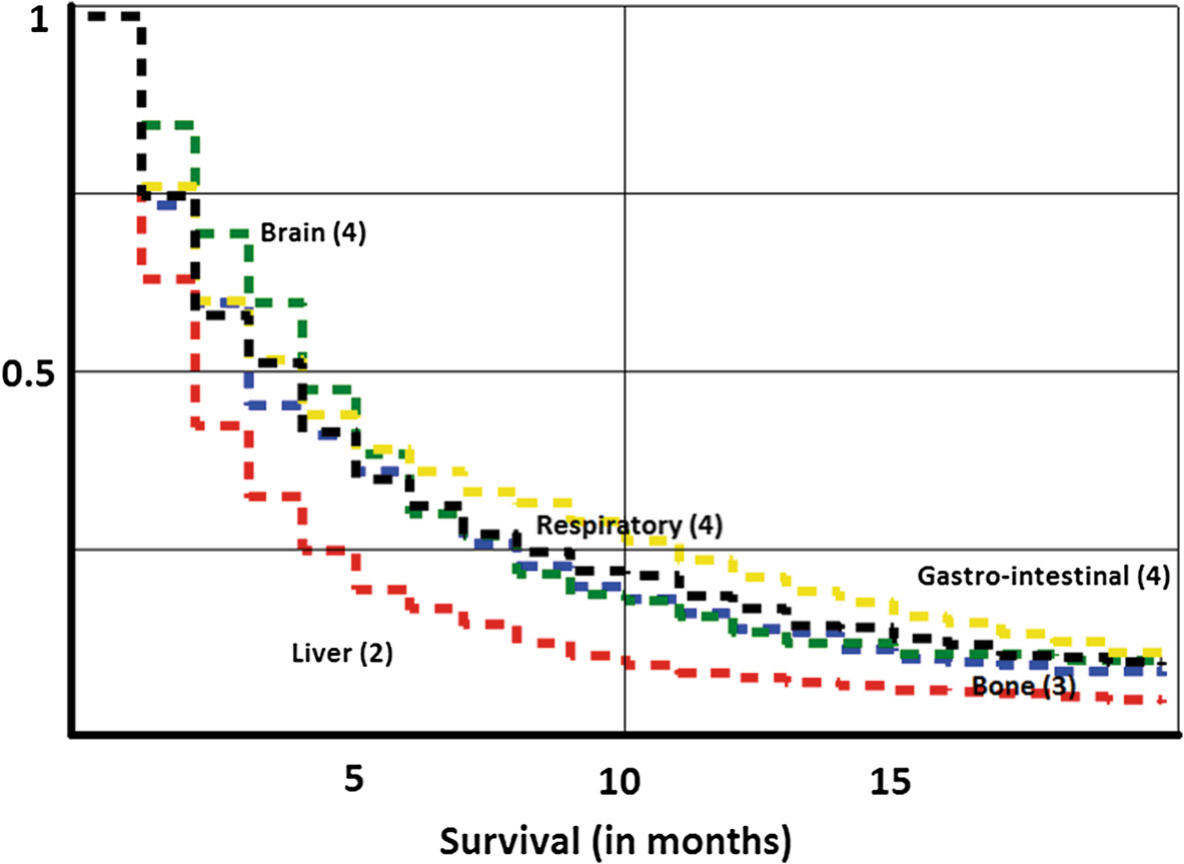
**Kaplan-Meier survival curves for death in CUP, depending on the site of CUP metastases.** Median survival times are displayed. Red = liver. Green = brain. Yellow = gastro-intestinal. Black = respiratory, Blue = bone.

A total of 158,670 patients with cancer of lung, colorectum, prostate, pancreas, stomach, breast, kidney, and bladder diagnosed between 2002 and 2008 were identified. Of these patients, 50,074 died during the study period, and 14,698 had metastatic cancer at diagnosis (M1). After excluding cancer patients with multiple metastases and deaths occurring within one month after diagnosis, 6,745 cancer patients were selected for the analyses. The reference group consisted of 2,881 CUP patients with a single metastasis. Of the included metastatic sites, survival was worst for CUP of the liver (Figure 1). Median survival times (in months) for the different locations of CUP were as follows: 4 for respiratory, other gastro-intestinal and brain; 2 for liver; and 3 for bone.

HRs for death were assessed, comparing metastatic cancers with a known primary location with CUP at corresponding locations. All patients with metastatic cancer were compared, irrespective of metastatic location (Table 1). Patients with metastatic cancer from a known primary had a decreased risk of death compared to patients with any CUP (HR = 0.69). Patients with metastatic pancreatic, liver, and stomach cancer had an increased risk of death compared with CUP patients with metastases at these locations, whereas patients with metastatic colorectal, kidney, breast, and prostate cancer had a decreased risk of death compared with CUP patients. Survival in CUP with lung metastases was similar to that in metastatic lung cancer.

**Table 1:** Risks of death for metastatic cancers compared to CUP (cancer of unknown primary) patients diagnosed with these metastases

**Primary site**	**N**	**HR**	**95% CI**	
*CUP (reference)*	2881	1		
*Colorectal cancer*	1438	**0.61**	0.57	0.65
*Pancreatic cancer*	460	**1.71**	1.54	1.90
*Stomach cancer*	322	**1.16**	1.02	1.31
*Liver cancer*	188	**1.58**	1.36	1.84
*Lung cancer*	2453	0.98	0.92	1.04
*Kidney cancer*	284	**0.71**	0.62	0.80
*Bladder cancer*	139	0.93	0.78	1.11
*Prostate cancer*	1259	**0.24**	0.22	0.26
*Breast cancer*	202	**0.53**	0.45	0.61
*All known primaries*	6745	**0.69**	0.66	0.72

Patients were diagnosed and died between 2002 and 2008. Only patients with one primary cancer and a positive metastasis status were considered.

Next, individual metastatic sites were analyzed (Table 2). The liver was the most common CUP metastasis site (983 cases). Patients with known primary cancers were at a decreased risk of death compared to those with CUP when the organ of metastasis was the liver (0.58), bone (0.57), or gastro-intestinal organs (0.78). Survival from respiratory and brain metastases was similar in patients with CUP and patients with known primaries. Patients with pancreatic liver metastases were found to be at an increased risk of death (1.24) compared to patients with CUP of the liver. However, patients with colorectal and lung cancer had decreased risks of death. Patients with metastatic lung cancer showed similar survival to CUP patients. The exception was patients with liver metastases, with lung cancer patients having a decreased risk of death compared to CUP patients. For most metastatic locations, colorectal, kidney, prostate, and breast cancer patients had a decreased risk of death compared with CUP patients with corresponding metastases. Bone metastases originating from the prostate seem to have exceptional prognosis compared with CUP of bone.

**Table 2:** Risks of death for metastatic cancers compared to CUP (cancer of unknown primary) patients diagnosed with these metastases

	**Site of metastasis**																			
	**Respiratory system**				**Gastro-intestinal system**				**Liver**				**Brain**				**Bone**			
**Primary site**	**N**	**HR**	**95% CI**		**N**	**HR**	**95% CI**		**N**	**HR**	**95% CI**		**N**	**HR**	**95% CI**		**N**	**HR**	**95% CI**	
*CUP (reference)*	286	1			878	1			983	1			131	1			215	1		
*Colorectal cancer*	38	**0.44**	0.30	0.64	46	**0.67**	0.49	0.93	587	**0.42**	0.37	0.46	11	**0.28**	0.13	0.60	6	0.80	0.32	2.04
*Pancreatic cancer*	21	1.61	0.92	2.81	11	0.75	0.38	1.47	235	**1.24**	1.06	1.46	2	5.69	0.70	46.11	3	0.75	0.17	3.19
*Stomach cancer*	6	**2.76**	1.01	7.54	23	1.51	0.94	2.45	110	0.98	0.78	1.23	2	1.45	0.21	9.77	12	0.88	0.38	2.03
*Liver cancer*	14	1.17	0.60	2.26	8	0.75	0.33	1.68	73	1.19	0.92	1.55	1	3.34	0.19	58.65	8	0.56	0.21	1.47
*Lung cancer*	71	1.36	0.99	1.87	10	0.68	0.36	1.32	166	**0.76**	0.63	0.90	574	1.00	0.81	1.23	317	0.99	0.81	1.22
*Kidney cancer*	45	**0.65**	0.44	0.97	0	0.00	0.00	0.00	11	**0.50**	0.26	0.96	7	0.72	0.24	2.17	31	0.81	0.50	1.30
*Bladder cancer*	18	0.83	0.46	1.50	1	7.70	0.83	71.62	7	1.44	0.65	3.19	5	0.64	0.15	2.65	20	0.95	0.52	1.71
*Prostate cancer*	8	0.42	0.13	1.37	1	0.11	0.01	0.90	14	**0.25**	0.14	0.45	6	0.48	0.11	2.16	481	**0.27**	0.21	0.34
*Breast cancer*	15	0.83	0.37	1.84	1	0.29	0.04	2.13	13	**0.31**	0.16	0.58	9	0.66	0.15	2.87	26	**0.50**	0.29	0.87
*All known primaries*	236	0.83	0.68	1.01	101	**0.78**	0.62	0.99	1216	**0.58**	0.53	0.63	617	0.95	0.78	1.17	904	**0.57**	0.48	0.69

Patients were diagnosed and died between 2002 and 2008. Only patients with one primary cancer and a positive metastasis status were considered.

**Figure 2:**
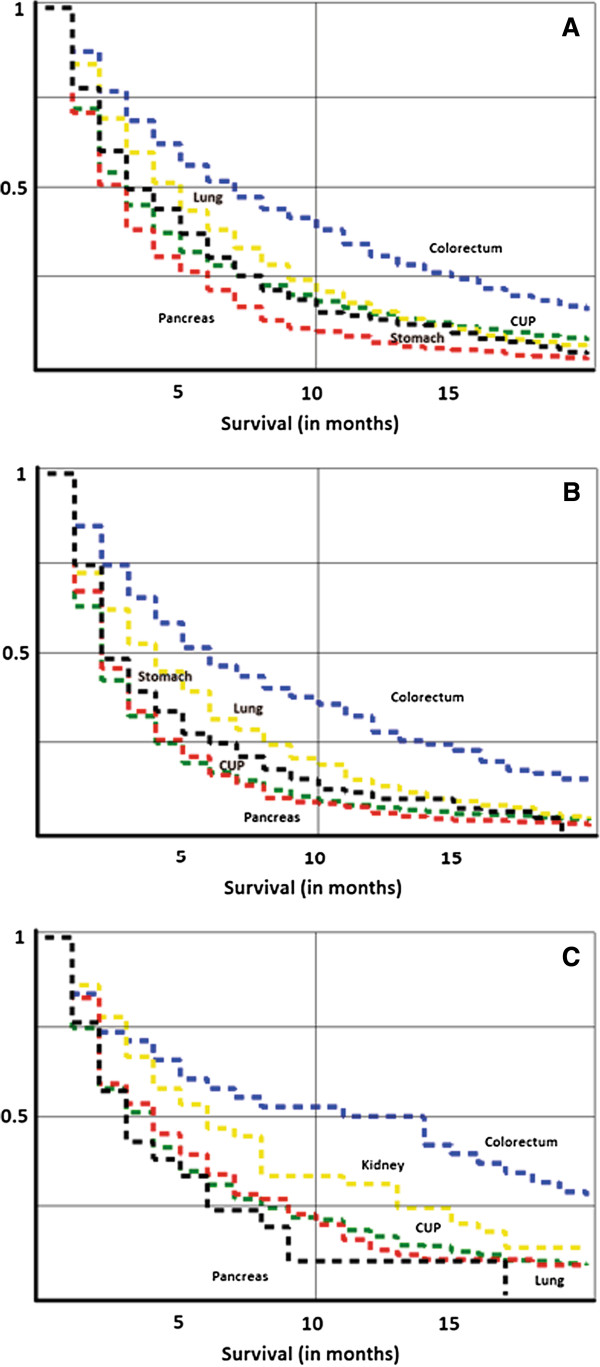
**Kaplan-Meier survival curves for death, depending on the primary site of metastatic cancer.** (**A**) Death in any metastatic location from indicated primary cancers or CUP, (**B**) death in liver metastases from indicated primary cancers or CUP, and (**C**) death in metastases to respiratory organs from indicated primary cancers or CUP.

The Kaplan-Meier plots in Figure 2 illustrate survival from metastatic cancer depending on the primary site. Among the indicated primary cancers and CUP, without regard to the metastatic location, survival was poor for pancreatic cancer compared with colorectal cancer (Figure 2A). Among the indicated primary cancers with liver metastases, lung and colorectal cancers showed better survival than CUP of liver (Figure 2B). In the case of respiratory metastases, colorectal and kidney cancer patients had better survival than patients with CUP (Figure 2C).

## Discussion

Population-based cancer registries are important sources of data for cancer control. However, most cancer registries lack data on the location of metastases and thus they have inherent weaknesses in following the process that eventually kills the patient. In this paper we present some alternative sources of data in order to address questions about survival depending on the location of metastasis. We investigated survival of patients with metastases from known primaries, compared with CUP patients, whose primaries were by definition unknown. Overall, survival in metastatic cancer was better if the primary location was known. However, metastatic pancreatic, liver, and stomach cancers –known for their poor prognosis- overall featured worse survival than CUP. The present results show 1) variations in risks of death in patients with defined metastases depending on the primary site of the malignancy, and that 2) survival in CUP is generally worse than with primary cancers metastatic to the same organ where CUP was detected. This may be due to the aggressive behavior of CUP. Although the primary tumor in CUP is thought to be dormant, CUP patients feature early distant metastases [15,18,19]. The metastatic tendency may explain the poor prognosis, and, as in primary cancers, metastases are thought to be the cause of death in most cancer patients [1,2,7]. Several genes have been implicated in metastasis [6,20,21]. Indeed, some important metastatic genes have been shown to be overexpressed in CUP: vessel endothelial growth factor, which induces angiogenesis [21], and matrix metalloproteinases, proteolytic enzymes mediating local invasion and metastasis [20].

The estimations of median survival times for included CUP patients, three months, is consistent with previous reports of approximately three to four months in population-based studies [8,15,22]. It has been earlier noted that some hospital-based studies have estimated substantially longer CUP survival, probably due to different inclusion criteria [22]. Although the prognosis of CUP is overall poor, some 15–20% of the patients present with less aggressive and/or treatable tumors of favorable prognosis [23]. Some of these include CUP diagnosed in lymph nodes only and others require clinical information not available in the present study, including some colorectal and breast cancers. The present metastatic sites would largely belong to the 80–85% of CUP on unfavorable prognosis. Although therapies have improved, particularly in the favorable subset, there has been no evidence that the overall survival would have changed, unfortunately alike many metastatic cancers [24,25]. Fast diagnosis is important in CUP and new methods include immunohistochemical and gene expression based methods for tissue-of-origin identification [23,26,27]. If the primary cancer can be identified the diagnosis is changed to that cancer which would not be scored as CUP in the present analysis. CUP incidence has been declining during the past decade in many countries and improved detection of primary cancers may have contributed to this trend [25].

Recently, patients with CUP of brain have been shown to have better survival (HR = 0.85/0.79 men/women)[8] compared to patients with known primaries. However, other investigators have not found any difference in brain metastasis survival between patients with a known or unknown primary location [16]. In the present study, no differences could be found. Lung cancer was the most common source of brain metastases in the present study. Thus, we speculate that the primary source of CUP of the brain may in fact often be lung cancer, which is also the most common cause of death in CUP patients [18].

In our dataset of CUP patients, CUP of liver featured the shortest median survival, only two months. The poor prognosis of liver involvement in CUP is known: previous estimates have ranged between 1.7 and 10 months [19,28]. Similarly, CUP patients with liver involvement have been shown to be at an increased risk of death (HR = 1.63) compared with CUP patients without liver involvement. Histological features consistent with neuroendocrine carcinoma have been associated with significantly better prognosis than adenocarcinoma. Liver metastases commonly arise from colorectal cancer, and the five year survival in patients not receiving surgery has been reported to be less than 5% [29]. More recently, the five year survival in selected groups receiving surgery may approach 50% [30]. The HR for colorectal cancer with liver metastasis was 0.42 but those from prostate (0.25) and breast (0.31) cancer were even more favorable. Pancreatic cancer, frequently featuring liver metastases, is associated with a dismal prognosis. Survival in patients with pancreatic liver metastases has been approximated to less than three months [31]. However, median survival in patients receiving surgery has been estimated to 11.4 months [32]. Novel developments regarding chemotherapeutic regimens in selected patients also show promising results [33].

The skeletal system has been described as the most common site for metastases [34]. Present results show that patients with skeletal lung cancer metastases had similar risks of death than CUP of bone. The prognosis among lung cancer patients with bone metastases was unfavorable. Previous results are in line with our findings, the median survival being only three months even in patients receiving surgical treatment [35]. Remarkably, the HR for prostate cancer was only 0.27 and those of kidney and breast cancer were 0.51 and 0.50, respectively.

Our study has several strengths. We used a national database, considered to be close to 100% complete in cancer registration [17]. Population-based studies on metastatic cancers may encounter problems regarding exclusion of the metastatic sites in the TNM-coding system. The Database used in the present study incorporates data from both the Swedish Cancer Registry and the Swedish Cause of Death Registry. Therefore, we could use the death certificates of cancer patients to identify the locations of metastases. The validity of death certificates in Sweden has a considerable impact on the reliability of our results. In Sweden, the proportion of deaths occurring in hospitals is very high. In 2003, 62.5% of deaths occurred in hospitals, whereas the rest occurred in other health care facilities (nursing homes, hospices etc.) or at home [13]. Furthermore, when considering only deaths with malignancies as the underlying cause, the proportion of hospital death has been shown to be as high as 85.1%. In hospital deaths, the issuing doctor of the death certificate is likely to have been involved in the treatment of cancer patients and therefore have insight in the patient’s history. Therefore, we believe that the high number of hospital deaths in Sweden strengthens the validity of death certificates. Also, the validity of death certificates with a malignancy as the underlying cause has been thought to be among the highest [12].

Can we be sure that the metastases mentioned in the death certificate were present at the time of diagnosis? This issue was addressed by only including cancer patients with positive distant metastatic status at diagnosis. Moreover, we excluded from our analyses decedents with more than one metastasis mentioned in their death certificate. Finally, in order to exclude the possibility that metastases might have been seeded from another primary site, we also excluded cancer cases with primary cancer diagnoses at multiple sites. Naturally, restriction of investigations to cancer patients with metastases at only one defined organ had a substantial impact on the number of cases available for analysis.

The poor survival in CUP may be due to its aggressive behavior. CUPs may undergo substantial phenotypic changes in order to avoid immunological surveillance, and the primary tumor may in fact reside in the same organ as the metastases themselves [18]. Among different sites, CUPs of the liver have the worst prognosis. It is tantalizing to speculate that this is linked to the immune hypothesis relating to CUP [18]. If many CUP cases are indeed due to prior immunological eradication of the primary, and CUP metastases thus represent immunological escape variants, it is perhaps logical that their growth is fastest in the liver, which has been proposed an “immune suppressive organ” [36]. Overall, the survival of CUP patients was shorter than with patients with known primaries. This is compatible with CUP representing tumors subjected to significant prior immunoediting and/or featuring a high degree of immunosuppression. A tumor initially sensitive to immunological control might have ample time to become more malignant through accumulation of hundreds of mutations during a prolonged equilibrium phase [37]. Upon escape from the equilibrium state an unstoppable killer is then unleashed. CUP accounts for 3–5% of cancer diagnoses, and although it is associated with a bad prognosis, some chemotherapeutic regimens have shown promising results [38-40]. Further research is motivated in order to increase understanding of this large group of cancer patients.

## Conclusions

CUP at many metastatic locations featured poorer prognosis than metastatic cancers with a known primary location and survival in CUP was comparable to that of metastatic lung cancer. As another novel finding, we showed large survival differences in patients with defined organ metastasis depending on the primary tumor.

## Competing interests

The authors declare that they have no competing interests.

## Authors’ contributions

Conception of the study: KH, MR, AH. Provision of data: KS. Data analysis: MR, KH, HT. Manuscript writing: MR, KH, AH. Approval of the final manuscript: all authors.

## Pre-publication history

The pre-publication history for this paper can be accessed here:

http://www.biomedcentral.com/1471-2407/13/36/prepub
